# Supplementary material for: Causal Relationship between Aging and Anorexia Nervosa: A White-Matter-Microstructure-Mediated Mendelian Randomization Analysis
Source: Biomedicines. 2024 Aug 16;12(8):1874. doi: 10.3390/biomedicines12081874 (PMC11351342; doi:10.3390/biomedicines12081874)
Supplement: Supplementary file 1 [file biomedicines-12-01874-s001.zip › Supplementary Figure/Figure S4/Singe SNP plot of MR analysis for TL to ubm-b-1511.pdf]

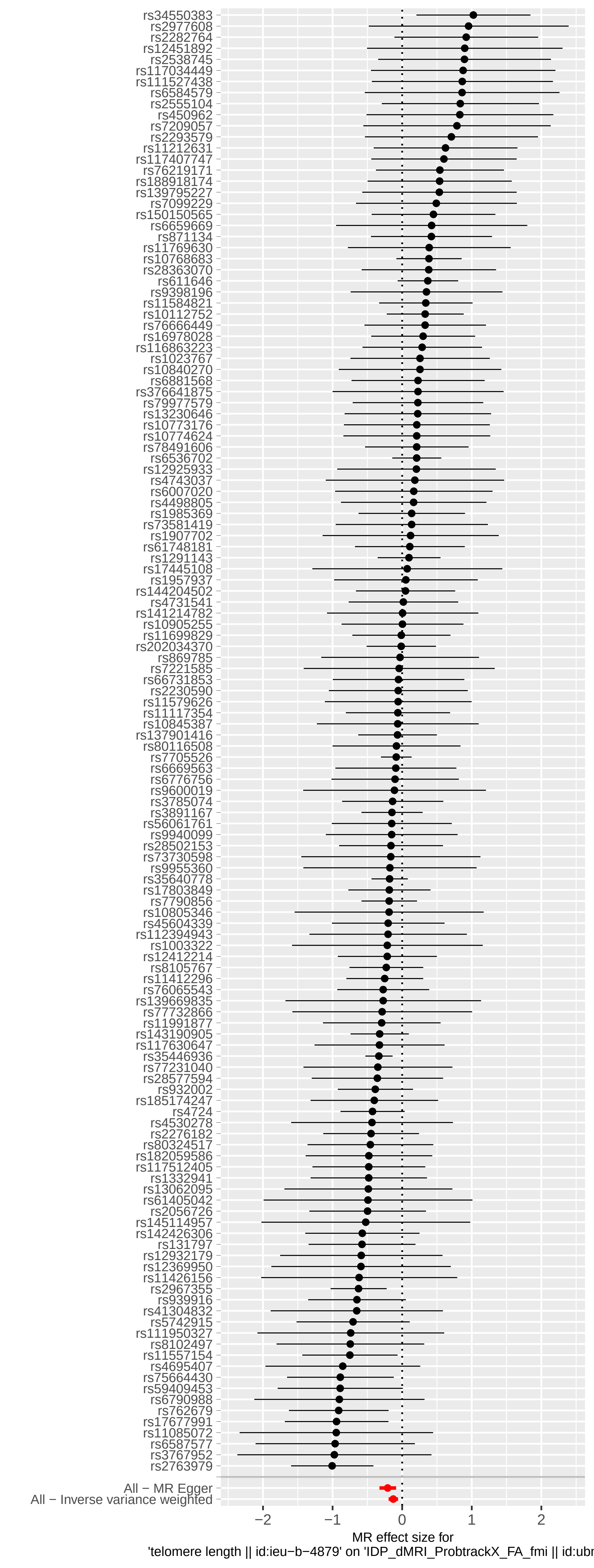
rs34550383
rs2977608
rs2282764
rs12451892
rs2538745
rs117034449
rs111527438
rs6584579
rs2555104
rs450962
rs7209057
rs2293579
rs11212631
rs117407747
rs76219171
rs188918174
rs139795227
rs7099229
rs150150565
rs6659669
rs871134
rs11769630
rs10768683
rs28363070
rs611646
rs9398196
rs11584821
rs10112752
rs76666449
rs16978028
rs116863223
rs1023767
rs10840270
rs6881568
rs376641875
rs79977579
rs13230646
rs10773176
rs10774624
rs78491606
rs6536702
rs12925933
rs4743037
rs6007020
rs4498805
rs1985369
rs73581419
rs1907702
rs61748181
rs1291143
rs17445108
rs1957937
rs144204502
rs4731541
rs141214782
rs10905255
rs11699829
rs202034370
rs869785
rs7221585
rs66731853
rs2230590
rs11579626
rs11117354
rs10845387
rs137901416
rs80116508
rs7705526
rs6669563
rs6776756
rs9600019
rs3785074
rs3891167
rs56061761
rs9940099
rs28502153
rs73730598
rs9955360
rs35640778
rs17803849
rs7790856
rs10805346
rs45604339
rs112394943
rs1003322
rs12412214
rs8105767
rs11412296
rs76065543
rs139669835
rs77732866
rs11991877
rs143190905
rs117630647
rs35446936
rs77231040
rs28577594
rs932002
rs185174247
rs4724
rs4530278
rs2276182
rs80324517
rs182059586
rs117512405
rs1332941
rs13062095
rs61405042
rs2056726
rs145114957
rs142426306
rs131797
rs12932179
rs12369950
rs11426156
rs2967355
rs939916
rs41304832
rs5742915
rs111950327
rs8102497
rs11557154
rs4695407
rs75664430
rs59409453
rs6790988
rs762679
rs17677991
rs11085072
rs6587577
rs3767952
rs2763979
All − MR Egger
All − Inverse variance weighted
−2
−1
0
1
2
MR effect size for
'telomere length || id:ieu−b−4879' on 'IDP_dMRI_ProbtrackX_FA_fmi || id:ubm
